# CABIF-Net: Robust Confidence-Based Audio-Visual Fusion for Fine-Grained Bird Recognition

**DOI:** 10.3390/biology15080661

**Published:** 2026-04-21

**Authors:** Zilong Li, Yan Zhang, Danju Lv, Yueyun Yu

**Affiliations:** 1 College of Big Data and Intelligent Engineering, Southwest Forestry University, Kunming 650224, China; lizilong@swfu.edu.cn (Z.L.); lvdanjv@swfu.edu.cn (D.L.); yuyueyun103@swfu.edu.cn (Y.Y.); 2 College of Science, Southwest Forestry University, Kunming 650224, China; 3 Yunnan Provincial Department of Education Key Laboratory of Mathematical and Physical Applications in Forestry, Southwest Forestry University, Kunming 650224, China

**Keywords:** bird species classification, fine-grained, multimodal classification, feature fusion

## Abstract

Fine-grained bird identification is crucial for ecosystem monitoring, species conservation, and habitat assessment. However, in real-world environments, there are challenges such as imbalances in modality quality and interference from background noise. To improve fine-grained audio-visual bird classification under heterogeneous modality conditions, we propose an audio-visual feature fusion framework named CABIF-Net. This framework introduces a confidence-based Top-K mean pooling module to select key frames to optimize the visual representations at the video level. Through a Confidence Calibration module, it dynamically assesses the reliability of the visual and audio modalities and integrates a Bidirectional Inter-modulation Fusion module to achieve controllable cross-modal information interaction. Experiments were conducted on the publicly available SSW60 dataset, characterized by severe noise and imbalance in modality quality, and the self-built Birds21 dataset with balanced modality quality. The experimental results show that the classification accuracies were 85.76% and 96.67%, respectively, outperforming existing unimodal methods and several mainstream fusion strategies. Weight distribution and visualization analyses further indicate that the proposed method can adaptively adjust the modality contributions based on discriminative evidence at the sample level. This study provides an effective framework for fine-grained audio-visual bird species recognition.

## Simple Summary

Fine-grained identification of bird species plays an important role in ecological monitoring, species conservation, and habitat assessment. However, automated methods often struggle in natural environments due to noise, varying recording conditions, and imbalances in the quality of visual and audio data. In this study, we develop a new multimodal fusion framework, CABIF-Net, that combines visual and audio information to improve the robustness and accuracy of bird species recognition. CABIF-Net selects the most informative video frames, dynamically evaluates the reliability of each modality, and adaptively integrates visual and audio signals. Experiments are conducted on two representative bird datasets, one of which contains noisy and imbalanced data and the other which has more balanced visual and audio quality. The results show that CABIF-Net consistently outperforms existing unimodal and multimodal fusion approaches. This study provides a framework for fine-grained audio-visual bird species recognition and may offer support for future research on multimodal bird monitoring under complex field conditions.

## 1. Introduction

Birds are sensitive to environmental changes and serve as the key indicator species of ecosystem health [1]. Their population dynamics are widely used to assess habitat quality, ecological stress factors, and the effectiveness of conservation actions [2]. Accurate bird species identification not only supports habitat assessment and population monitoring but also provides a basis for prioritizing conservation measures and analyzing long-term ecological trends [3]. However, automated bird identification remains challenging in complex natural environments. Misclassification can lead to deviations in species distribution and population estimates, and weaken the scientific rigor of ecological decision-making monitoring data [4,5]. Therefore, in ecological applications involving bird monitoring tasks, it is crucial to improve the accuracy and robustness of automated bird species recognition [6].

In recent years, deep learning has provided a new technical foundation for automatic identification of birds in visual and acoustic domains [7]. In the acoustic modality, discriminative features are typically extracted by converting the raw audio into time–frequency representations [8], such as spectrograms and cepstral coefficients, and then classified using convolutional neural networks (CNNs), convolutional recurrent neural networks (CRNNs) [9], or models based on the Transformer [10]. In end-to-end learning, learnable front ends such as SincNet and LEAF can directly learn filtering and compression operations from waveforms, which demonstrates stronger cross-domain generalization ability in cases of limited samples [11]. In the visual modality, the main research direction is object detection and fine-grained classification [12]. Object detection methods based on YOLO can reliably locate the body of birds in chaotic backgrounds [4], while models based on Vision Transformer (ViT) enhance the fine-grained discriminative ability through a global attention mechanism [13]. Although large self-supervised and Transformer models perform well in standard benchmarks, their high computational costs and data requirements limit their practical application scope [14]. Transferable CNN models, such as ResNet [15] and other modern CNN backbones [16], are widely adopted due to their mature feature extraction capabilities and efficient computational performance.

However, in real-world ecological monitoring environments, unimodal methods still have significant limitations [17]. In the acoustic field, background noise, overlapping vocalizations, and changes in recording equipment reduce the detectability of target sound sources and decrease generalization across different scenarios [18]. In the visual field, small target size, occlusions, complex backgrounds, and the morphological similarity among closely related species further increase the difficulty of detection and classification [19]. Furthermore, false positives and false negatives from unimodal recognition can introduce systematic biases in the estimation of species occupancy rates and activity patterns [5]. These challenges increasingly constrain the performance improvement of unimodal methods in noise suppression, distinction of similar species, and cross-domain robustness.

To overcome the limitations of unimodal methods in complex environments, researchers have explored the integration of multimodal information to improve recognition performance. Feature-level fusion typically involves combining different time–frequency views or multi-scale features to enhance discriminative ability. The EMSCNN model, proposed by Liu et al., employs multi-scale convolutional fusion on wavelet spectrograms and achieved an accuracy of 91.49% in 30-species bird recognition experiments [20]. This indicates that multi-scale feature fusion can enhance the discriminative ability of acoustic modality. Another approach strengthens representational ability through cross-feature family fusion. For example, Yang et al. propose SSL Net, which combines handcrafted spectral features with learned deep representations, and compared different feature-level fusion strategies. On the Western Mediterranean Wetland Birds (WMWB) dataset, compared with the accuracy of the single optimal branch at 84.02%, feature fusion increased accuracy to 87.29% (Spectral+LEAF, shared) and 86.54% (Spectral+BEATs, shared), with an F1 score of up to 88.79, thereby verifying the effectiveness of the fusion strategy [21]. The cross-modal fusion methods, which combine visual and audio information, can further improve performance. In earlier research, Zhang et al. (AAAI 2018) introduce dual-branch image and audio encoders and employ an attention mechanism to learn audio-visual representations; on the CUB-200-2011 dataset, this approach increased the Top-1 accuracy of ResNet-50 from 63.25% to 69.8% only using visual input, demonstrating the effectiveness of audio-visual fusion in fine-grained recognition [22]. Naranchimeg et al. compared early, mid-term, and late fusion strategies, and found that a two-stage fine-tuning method with element-wise summation at the late fusion stage achieved a 78.9% classification accuracy on the CUB-200-2011 [23]. This result surpassed that of all unimodal baselines and alternative fusion configurations. To support multimodal learning in real scenarios, Van Horn et al. released the SSW60 dataset. Its official benchmark reached a Top-1 accuracy of 80.6% on the SSW60 video test set using score-level late fusion, indicating the potential of audio-visual fusion on complex ecological data [24]. To address the imbalance of audio-visual features, Xu et al. propose a fusion strategy based on a multimodal cosine loss to jointly regularize features and fusion weights of specific modalities. On the SSW60, the mid-level concatenation increased classification accuracy from approximately 73.5% to 75.9%, further verifying the importance of optimizing fusion mechanisms [25]. More recent work, such as the MSFG-AVFNet proposed by Xie et al., designed a multi-stage fine-grained feature extraction and a robust fusion mechanism, achieving a Top-1 accuracy of 85.14% on SSW60, further confirming the positive impact of cross-modal fusion on recognition performance [26]. Apart from the specific audio-visual recognition studies for birds, the representative multimodal architectures discussed in the literature also explore more structured fusion patterns. For example, GlocalNet adopted a two-stage global-to-local framework to simulate rough global dependencies, and refined them into a more dense representation [27]. Hierarchical multimodal models can organize audio-visual information across multiple semantic levels and gradually integrate complementary cues from different levels, which improves the robustness of multimodal representations [28]. In addition, methods based on transformers, such as MulT, introduce directional pairwise cross-modal attention to capture long-range interactions between unaligned multimodal sequences [29]. These studies show that structured cross-modal modeling can improve multimodal representation learning by more effectively organizing and integrating complementary information from different modalities. Overall, existing studies demonstrate that well-designed fusion strategies can mitigate modal noise and category confusion. However, under more challenging conditions, such as wild ecological monitoring, for adapting to fluctuations in modality quality, and evaluating reliability at the sample level, further research is still needed.

Although the latest advances in cross-modal fusion methods have improved complementarity, they still face significant challenges in real-world ecological monitoring conditions. On one hand, in natural environments, factors such as occlusions, cluttered backgrounds, sensor differences, and environmental noise can cause substantial fluctuations in the quality of visual and audio modalities, making cross-modal alignment and discriminative complementarity more difficult [30]. On the other hand, most existing fusion methods do not explicitly consider the differences in modality quality and changes in sample-level reliability, which makes it difficult to effectively regulate cross-modal information interaction under low-quality or distorted data conditions, reducing discriminative robustness [31]. Therefore, developing an audio-visual fusion mechanism that can comprehensively assess the reliability of each modality and fully exploit complementary information remains a key challenge for improving the accuracy and robustness of multimodal bird species recognition in ecological monitoring.

To fully exploit the potential of audio-visual multimodal information and develop a cross-modal fusion mechanism, which can evaluate the reliability of each modality and utilize complementary information, we propose a confidence-based audio-visual feature-fusion network, named CABIF-Net. This method assesses the reliability of different modalities through dynamic weight adjustment, and combines key-frame selection with feature-level fusion to obtain controllable interaction between visual and audio information, enabling the fusion system to extract distinctive features more robustly in complex natural environments. Specifically, this study designs a frame-level confidence-based selection strategy, a Confidence Calibration module, a Bidirectional Inter-modulation Fusion mechanism, and an end-to-end optimization pipeline. The main contribution of this work is the development of a robust multimodal recognition framework for automated bird species identification in complex environments. Experimental results show that CABIF-Net significantly outperforms unimodal methods and several mainstream fusion strategies on the SSW60 dataset with noise and modality imbalance, as well as the Birds21 dataset with balanced modality. Overall, this study establishes a framework for fine-grained audio-visual bird species recognition under heterogeneous modality conditions, with ecological monitoring considered as a potential application background.

The main contributions of this work are summarized as follows:

(1) Propose an audio-visual fusion framework, CABIF-Net, for fine-grained bird recognition. This framework can effectively address challenges in real-world scenarios, such as modality quality imbalance and noise interference. The CABIF-Net assesses the reliability of modalities at the sample level and realizes controllable feature interactions, resulting in more robust and reliable multimodal discrimination.
(2) Design a frame-level confidence-based Top-K mean pooling module, which prioritizes the aggregation of the most discriminative visual frames, significantly reducing the interference of redundant and noisy frames in video data, and improving the quality of video-level visual representation.
(3) Introduce a unified Confidence Calibration and Bidirectional Inter-modulation Fusion module, which explicitly evaluates the reliability of visual and audio modalities through temperature scaling and power-law calibration. By dynamically adjusting cross-modal interactions at the feature level, this mechanism enables high-quality modalities to dominate during the fusion process while retaining complementary contributions of weaker modalities, thereby improving discrimination performance and robustness.

## 2. Materials

In this study, the proposed method was evaluated on two representative datasets: the publicly available SSW60 (https://github.com/visipedia/ssw60, accessed on 4 February 2026) and the self-built Birds21. To clearly illustrate the differences in details between these two datasets, Table 1 summarizes their category sizes, modality composition, training and testing divisions, and data sources, making the experimental setup and subsequent result interpretation more intuitive and comparable.

**Figure 1:**
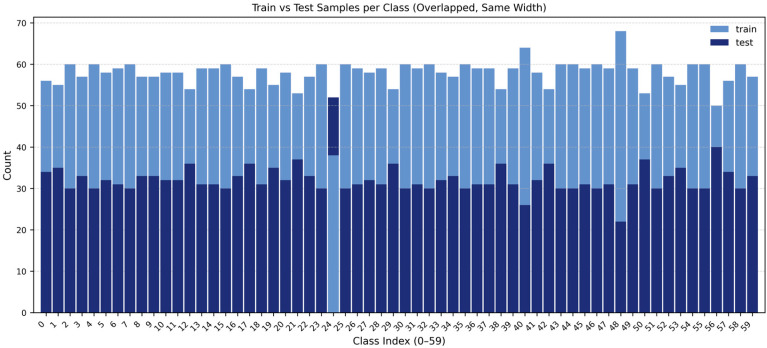
Illustration of the training and test sets in the SSW60 dataset.

The SSW60 is a publicly available fine-grained audio-visual bird dataset that covers 60 species, and consists of 5400 video clips each lasting 10 s. All clips were recorded in the wild and contain rich visual and acoustic information. The dataset includes pre-defined training and testing sets, and the species distribution is shown in Figure 1. To support pre-training and evaluation of the audio path, SSW60 also provides 3861 unpaired 10 s audio samples from the same species. To enhance the training of the visual path, the SSW60 also comes with a collection of static images corresponding to the 60 species, including 21,600 high-quality images from NABirds [32] and 10,221 relatively lower-quality images from iNat2021 [33], which are used for visual pre-training and cross-modal fusion learning.

Birds21 is a cross-modal image–audio dataset constructed in this study from public avian media repositories, covering 21 species of birds. The complete list of species can be found in Table A1 of Appendix A. This dataset was designed as a supplementary reference to the public SSW60 dataset, and its main purpose is to examine whether the proposed framework remains effective when the quality of the visual and audio modalities is more balanced. The images were collected from the Macaulay Library (https://www.macaulaylibrary.org/, accessed on 4 February 2026), and the audio recordings were obtained from (https://www.xeno-canto.org/, accessed on 4 February 2026). These 21 species were selected according to the availability of two modal sample types and sufficient sample size. The image and audio samples were also subjected to basic quality screening to remove severely degraded or unusable data.

To support multimodal learning, the two modalities were paired at the species level, rather than collected from the same observation or recording event. The dataset contains 17,207 images and 1186 audio recordings, with a total audio duration of 76,477.8 s. For each species, the dataset was divided into training and test sets at a ratio of 8:2.

## 3. Methods

**Figure 2:**
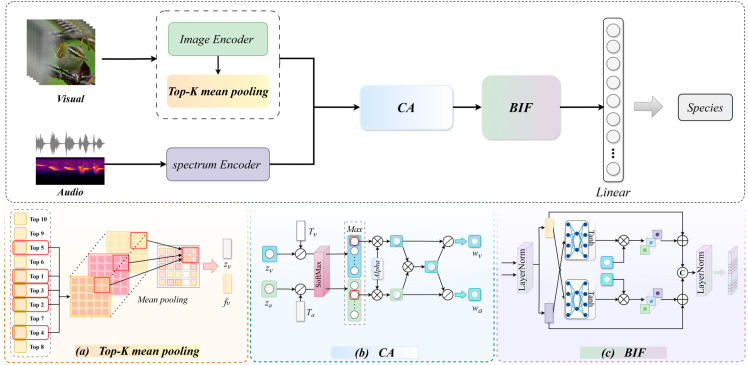
Technical pipeline of CABIF-Net. The upper part presents the overall workflow, while the lower part provides the detailed structures of the three key modules. Specifically, (**a**) illustrates the Top-K mean pooling module, (**b**) shows the Confidence Calibration (CA) module, and (**c**) depicts the Bidirectional Inter-modulation Fusion (BIF) module. (Bird image from iNaturalist photos 230127186 by Thompson Hyggen (CC0) https://www.inaturalist.org/photos/230127186, accessed on 10 February 2026).

In this study, we propose CABIF-Net, a confidence-based audio-visual fusion network that incorporates Confidence Calibration and Bidirectional Inter-modulation Fusion modules, aiming to address issues such as cross-modal complementarity, noise suppression, and the imbalance of modality quality in fine-grained bird classification. The framework comprises audio-visual feature extraction and a sample-level Confidence Calibration-based Bidirectional Inter-modulation Fusion module. The overall framework is illustrated in Figure 2. CABIF-Net processes the visual and audio modalities simultaneously and performs feature fusion through three key modules. Firstly, the visual branch extracts frame-level representations from the input image or sampled video frames. For video inputs, the visual branch further introduces a Top-K mean pooling module to select the k most discriminative frames and aggregate them into a video-level visual representation as well as corresponding visual logits. Meanwhile, the audio branch encodes the spectrogram of the Log Mel-filter bank obtained from the audio signal to extract global audio features and audio logits. Subsequently, the Confidence Calibration (CA) module takes the logits of the visual and audio modalities as inputs, calibrates their prediction confidence, and converts them into normalized modality weights that reflect sample-level reliability. Finally, the Bidirectional Inter-modulation Fusion (BIF) module uses these calibrated weights to regulate the bidirectional cross-modal feature interaction, allowing the more reliable modality to dominate the fusion process while retaining the complementary information provided by the weaker modality. The final fusion representation is then fed into a lightweight linear classification head to generate the final species prediction result.

### 3.1. Audio-Visual Feature Extraction

During the fusion process, in order to establish a fair and interpretable baseline and minimize the impact of encoder capacity differences, this study uses symmetric ResNet-18 backbones for visual and audio feature extraction [34]. Through global average pooling, the network generates 512-dimensional visual feature $f_{v}\in R^{512}$ and audio feature $f_{a}\in R^{512}$. By keeping the encoder design and capacity consistent, this symmetric setup controls the encoder differences as a constant, allowing the observed performance to more directly reflect intrinsic modal properties and the effectiveness of the fusion mechanism, thereby providing a reliable basis for objective evaluation.

#### 3.1.1. Audio Feature Extraction

Audio processing uses ResNet-18 as its backbone, initialized with pre-trained weights related to AudioSet to enhance general acoustic feature representation. To make the model suitable for bird vocalization classification, the audio encoder is first fine-tuned on the target dataset, and then it is frozen to keep its feature extraction capability consistent with the visual path. During the preprocessing, the raw audio is resampled at 16 kHz mono and converted to a Log Mel-filterbank spectrogram for network input. Specifically, the waveform is transformed to the time–frequency domain using a short-time Fourier transform with a 1024-sample window and a 320-sample hop, mapped to 64 Mel bands spanning 50–8000 Hz, and converted to logarithmic amplitude to emphasize the energy distribution. Then, the time axis is adjusted to 501 frames through center cropping or zero padding, resulting in a normalized 64 × 501 spectrogram. Finally, this preprocessed spectrogram is fed into the audio encoder to produce a 512-dimensional global audio feature $f_{a}$.

#### 3.1.2. Frame-Level Visual Feature Extraction

For visual feature extraction, a ResNet-18 backbone initialized with pre-trained weights from ImageNet is used to obtain general visual representations. To adapt to the fine-grained bird recognition task, the encoder is fine-tuned on the target dataset, and then frozen to reduce overfitting and stabilize the feature space. The input images are resized to 224 × 224 pixels, normalized using the mean and standard deviation of ImageNet, and processed through the visual encoder and global average pooling to generate a 512-dimensional frame-level visual feature $f_{v}$.

#### 3.1.3. Video-Level Visual Feature Extraction and Top-K Mean Pooling

For video data, a Top-K mean pooling module is introduced in the visual feature extraction process to generate video-level visual representations, as shown in Figure 2a. This module aims to suppress the influence of redundant and noisy frames while retaining the most distinctive key frame information. Specifically, 10 frames are uniformly selected from each video clip and passed through the visual feature extractor to obtain 512-dimensional frame-level visual features $f_{v_{i}}$. Then, each frame representation $f_{v_{i}}$ is fed into the frozen visual classifier to produce frame-level logits $l_{i}\in R^{C}$, where C denotes the total number of classes. The logits $l_{i}$ is converted into the class probability distribution of each frame using the Softmax function, as expressed in Equation (1).

(1) $$p_{i,j}=\mathrm{Softmax}{\left(l_{i}\right)}_{j}=\frac{\exp\left(l_{i,j}\right)}{\sum_{m=1}^{C}\exp\left(l_{i,m}\right)}$$

Here, $p_{i,j}$ represents the probability of the i-th frame belonging to the j-th class, and m is the class index variable. Based on this distribution, the maximum class probability is taken as the confidence score $c_{i}$ of the frame to measure its discrimination ability, as shown in Equation (2).

(2) $$c_{i}=\underset{j=1,2,\ldots ,C}{\max}p_{i,j}$$

The higher the value of $c_{i}$, the more discriminative information the frame contains, and the stronger resilience to noise is. Frames are sorted according to their $c_{i}$ values, and then the top k frames with the highest confidence scores (in this work, k=5) are selected to form the key frame set S, as defined in Equation (3).

(3) $$S=\{i_{1},i_{2},\ldots ,i_{k}|c_{i_{1}}\geq c_{i_{2}}\geq \ldots \geq c_{i_{k}}\geq c_{j},\forall j\notin S\}$$

Here, $i_{1},i_{2},\ldots ,i_{k}$ are the indices of the selected key frames, and $c_{i_{1}},c_{i_{2}},\ldots {,c}_{i_{k}}$ are their respective confidence scores. To simplify the calculation process, the indices of the top k high-confidence frames are directly obtained via a Top-K operation, as shown in Equation (4).

(4) $$S=\mathrm{Top}-K\left(\{c_{i}{\}}_{i=1}^{N},k\right)$$

Here, N represents the total number of frames in the input video. Subsequently, the frame-level features in the set S are aggregated through mean pooling to obtain the video-level visual feature ${\overset{ˉ}{f}}_{v}$. Similarly, the frame-level logits $z_{v}$ is obtained, as formalized in Equations (5) and (6).

(5) $${\overset{ˉ}{f}}_{v}=\frac{1}{k}\sum_{i\in S}f_{v_{i}}$$

(6) $$z_{v}=\frac{1}{k}\sum_{i\in S}l_{i}$$

This Top-K mean pooling module preserves the most discriminative key frames through confidence-based selection and mean aggregation, while reducing the influence of low-quality frames on the video-level representation. The resulting video-level visual representation ${\overset{ˉ}{f}}_{v}\in R^{512}$ has the same dimension as the global audio feature, providing a unified and highly discriminative input for subsequent cross-modal fusion and classification.

### 3.2. Confidence Calibration and Bidirectional Inter-Modulation Fusion Module

To tackle common issues in audio-visual fusion, such as modality imbalance in quality and the adverse impact of noisy modalities on discrimination, we propose a Confidence Calibration and Bidirectional Inter-modulation Fusion module. This module consists of two main steps. Firstly, the logits from the visual and audio feature extractors are calibrated to produce sample-level modal weights, which quantify the relative reliability of each modality for a given sample. Secondly, these weights are used at the feature level to control the intensity of cross-modal adjustments during bidirectional inter-modulation, allowing the more reliable modality to dominate the fusion process and suppressing noise propagation from a less reliable modality, thereby enhancing the overall discriminative performance.

#### 3.2.1. Confidence Calibration and Weight Generation

To alleviate the problem of over-confidence in the original logits and explicitly quantify the reliability differences between the two modalities at the sample level, a Confidence Calibration (CA) module is introduced, as illustrated in Figure 2b. This module takes logits $z_{v}$ from the visual feature extraction and $z_{a}$ from the audio branch as inputs. Firstly, it uses temperature scaling [35] to calibrate the prediction distributions of each modality, then employs a power-law transformation [36] to adjust confidence differences, and finally outputs the sample-level weights $w_{v}$ and $w_{a}$. Temperature scaling is a probability calibration technique that adjusts the “sharpness” of the prediction distribution without changing the class ranking, thereby reducing the weight allocation bias caused by over-confidence. The module contains three learnable scalar parameters $T_{v}$, $T_{a}$, and α, which are all initialized to 1 and optimized through backpropagation during the end-to-end training process. Specifically, $T_{v}$ and $T_{a}$ are used to adjust the smoothness of the visual and audio probability distributions, respectively, while α controls the amplification or compression of the modality weight differences.

Firstly, temperature scaling is applied to the logits of the two modalities, and the calibrated class probability distribution is obtained through the Softmax function, as shown in Equation (7).

(7) $$p_{v}=SoftMax\left(\frac{z_{v}}{T_{v}}\right),\;p_{a}=SoftMax\left(\frac{z_{a}}{T_{a}}\right)$$

Here, $p_{v}$ and $p_{a}$ represent the probability distributions of the visual and audio modalities, respectively. When T>1, the distribution becomes smoother and the prediction is more conservative; when T<1, the distribution becomes sharper and the prediction is more confident. On this basis, the maximum class probability of each modality is taken as the prediction confidence of the current sample, as calculated in Equation (8).

(8) $$c_{v}=max\left(p_{v}\right),\;c_{a}=max\left(p_{a}\right)$$

Here, $c_{v}$ and $c_{a}$ represent the confidence of visual and audio modalities, respectively. Higher values indicate greater concentration in the prediction distribution of the corresponding modality, therefore reflecting higher reliability and stronger discriminative capability. To map the confidence of different modalities to the weights for fusion, the coefficient α is used to perform a power-law transformation on the confidence of the two modalities, and the result is normalized to obtain the visual weight, as shown in Equation (9).

(9) $$w_{v}=\frac{c_{v}^{\alpha }}{c_{v}^{\alpha }+c_{a}^{\alpha }}$$

Then, the audio weight is derived from the complementary relationship, as shown in Equation (10).

(10) $$w_{a}=1-w_{v}$$

When α>1, the modality with higher confidence is more likely to obtain a larger weight, strengthening the dominant position of the high-quality modality. When α<1, the weight distribution becomes more uniform, which helps retain complementary information as the confidence levels of the two modalities are similar.

#### 3.2.2. Bidirectional Inter-Modulation Fusion Module for Audio-Visual Features

The imbalance in modality brings about two main issues. On one hand, noise from low-quality modalities may spread during the fusion process, thereby contaminating the discriminative features of high-quality modalities. On the other hand, when cross-modal interaction is insufficient or limited to one-way adjustment, the model may fail to fully exploit complementary information of audio-visual features, and even may weaken the discriminative characteristics of a specific modality. To address these problems, a Bidirectional Inter-modulation Fusion (BIF) module is proposed for audio-visual features, as illustrated in Figure 2c. This module uses sample-level weights to control the intensity of cross-modal adjustment, allowing the high-reliability modalities to dominate the interaction while suppressing the adverse effects of low-reliability modalities, enhancing the quality and robustness of the fused features.

The input of this module is the visual feature $f_{v}$ and the audio feature $f_{a}$, each with a dimension of D=512. For the video input, $f_{v}$ represents the aggregated visual feature at the video level. The module also receives the sample-level weights $w_{v}$ and $w_{a}$, obtained in Section 3.2.1. The output is the fused feature $\phi \in R^{2D}$. When D=512, ϕ is a 1024-dimensional vector. Firstly, the two modal features are subjected to layer normalization to reduce the distribution differences and improve training stability, as shown in Equation (11).

(11) $$f_{v,n}=\mathrm{LayerNorm}\left(f_{v}\right),\;f_{a,n}=\mathrm{LayerNorm}\left(f_{a}\right)$$

$f_{v,n}$ and $f_{a,n}$ represent the normalized visual and audio features, respectively, while LayerNorm represents the layer normalization operation. Based on these normalized features, two lightweight MLPs are used to generate cross-modal modulation signals, and the modulation amplitude is limited within [−1, 1], which is achieved through the tanh (⋅) activation function to avoid excessive modulation. The modulation signal is defined as in Equation (12).

(12) $$s_{v}=\tanh\left({\mathrm{MLP}}_{a2v}\left(f_{a,\mathrm{nr}}\right)\right),\;s_{a}=\tanh\left({\mathrm{MLP}}_{v2a}\left(f_{v,\mathrm{nr}}\right)\right)$$

Here, $s_{v}$ and $s_{a}$ refer to the modulation signals for visual and audio modalities, respectively; ${MLP}_{a2v}$ and ${MLP}_{v2a}$ correspond to the lightweight multi-layer perceptrons in the conversion process from audio to visual, and from visual to audio. Each structure is as Linear (D, H)->SiLU->Linear (H, D). During the inter-modulation process, the modulation signals are injected as residuals into the corresponding modality representations, and modulation intensity is dynamically controlled by the confidence weights as described in Section 3.2.1. This enables the stronger modality to correct the weaker ones and achieve precise gating, where high-quality modality dominates and low-quality modality is regulated, resulting in efficient interaction of the bimodal features, as shown in Equation (13).

(13) $$f_{v,\mathrm{cm}}=f_{v,n}+w_{v}⊙s_{a},\;f_{a,\mathrm{cm}}=f_{a,n}+w_{a}⊙s_{v}$$

$f_{v,\mathrm{cm}}$ and $f_{a,\mathrm{cm}}$ are the modulated bimodal features, and ⊙ denotes element-wise multiplication. These two enhanced features are concatenated along the channel dimension and subjected to another layer normalization to obtain the fused feature, as shown in Equation (14).

(14) $$\phi =\mathrm{LayerNorm}\left(\mathrm{Concat}\left(f_{v,\mathrm{cm}},f_{a,\mathrm{cm}}\right)\right)$$

The fused feature ϕ is fed into a lightweight linear classification head to generate the fused logits vector $z_{fuse}$, as shown in Equation (15). The class with the highest score is selected as the final predicted label $\hat{y}$, as shown in Equation (16).

(15) $$z_{fuse}=LinearHead\left(\phi \right)$$

(16) $$\hat{y}=argmax\left(z_{fuse}\right)$$

This module works in coordination with the CA module introduced in Section 3.2.1, forming a two-layer fused architecture consisting of sample-level calibration and feature-level inter-modulation. The former determines the strength of cross-modal interaction, while the latter gives a dominant role to the more reliable modality during the interaction process. This design effectively reduces the risk of noise propagation caused by the imbalance of modality quality, while maintaining controllable computational efficiency. The fused features retain fine-grained visual discriminative cues and integrate audio time–frequency contextual information, providing a more reliable input representation for subsequent classification tasks and enhancing the robustness of the model in complex real-world field environments.

### 3.3. Classification Loss

To achieve effective parameter updates and improve classification accuracy, the cross-entropy loss is employed as the classification objective to optimize prediction based on the fused features. Cross-entropy measures the difference between the predicted distribution of the model and the true label distribution by minimizing the negative log-likelihood value corresponding to the correct labeled class. This enables the model to generate more accurate probability assignments, which is suitable for fine-grained bird classification. Its formula is depicted in Equation (17).

(17) $$L_{c}=-\sum_{c=1}^{C}y_{c,k}\cdot \log\left(p_{c,k}\right)$$

Here, C denotes the total number of classes, $y_{c,k}$ refers to the true label of the k-th sample in class c (one-hot encoding taking values 0 or 1), and $p_{c,k}$ is the predicted probability that the k-th sample belongs to class c. During the training process, the model is optimized end-to-end by minimizing $L_{c}$, thereby enhancing the separability of the fused representation and improving the performance of fine-grained classification.

To sum up, the process of CABIF-Net is depicted in Algorithm 1.
**Algorithm 1** CABIF-Net Inference ProcedureInput: sample (V,A), label y, Top-K k=5, class count COutput: predicted label $\hat{y}$1: Initialize frozen visual encoder $E_{v}$, frozen audio encoder $E_{a}$, frozen visual classifier $C_{v}$, frozen audio classifier $C_{a}$, confidence calibrator $\left(T_{v},T_{a},\alpha \right)$, modulation MLPs $\left({MLP}_{a2v},{MLP}_{v2a}\right)$, linear head Head(⋅), learned fusion parameters $\Theta_{fuse}$, epochs E, optimizer Opt2: for epoch = 1 to E do3:  for each mini-batch (V,A,y) do4:   // Step 1: Visual features with Top-K frame selection (for images, treat k=1)5:   For each frame $I_{i}$: $f_{v_{i}}=E_{v}(I_{i}),l_{i}=C_{v}(f_{v_{i}})$6:   Frame confidence: $c_{i}=max\left(Softmax(l_{i})\right)$7:   Select key frames: $S=Top-K(\{c_{i}{\}}_{i=1}^{N},k)$8:   Aggregate clip-level representation: $z_{v}={mean}_{i\in S}(l_{i}),\;{\overset{ˉ}{f}}_{v}={mean}_{i\in S}(f_{v_{i}})$9:   // Step 2: Audio features10:   $f_{a}=E_{a}(A),\;z_{a}=C_{a}(f_{a})$11:   // Step 3: Confidence Calibration 12:   $p_{v}=Softmax\left(\frac{z_{v}}{T_{v}}\right),p_{a}=Softmax\left(\frac{z_{a}}{T_{a}}\right)$13:   $c_{v}=max(p_{v}),c_{a}=max(p_{a})$14:   $w_{v}=\frac{c_{v}^{\alpha }}{c_{v}^{\alpha }+c_{a}^{\alpha }},w_{a}=1-w_{v}$15:   // Step 4: Bidirectional Inter-modulation Fusion16:   $f_{v,n}=LayerNorm(f_{v}),\;f_{a,n}=LayerNorm(f_{a})$17:   $s_{v}=tanh\left({MLP}_{a2v}(f_{a,n})\right),\;s_{a}=tanh\left({MLP}_{v2a}(f_{v,n})\right)$18:   $f_{v,cm}=f_{v,n}+w_{v}⊙s_{a}$19:   $f_{a,cm}=f_{a,n}+w_{a}⊙s_{v}$20:   // Step 5: Classification21:   $\phi =LayerNorm\left(Concat(f_{v,cm},f_{a,cm})\right)$22:   $z_{fuse}=LinearHead(\phi )$23:   $L_{c}=CrossEntropy(z_{fuse},y)$24:   update $\Theta_{fuse}{,T}_{v},T_{a},\alpha$ using Opt to minimize $L_{c}$25:  end for26: end for27: $\hat{y}=arg\;max(z_{fuse})$

## 4. Experiments and Result Analysis

To validate the effectiveness and generalizability of the proposed method, experiments were conducted on two datasets, Birds21 and SSW60. For Birds21, it was split into a training set and a test set at a ratio of 8:2. On these two datasets, we evaluated the effectiveness of the Top-K mean pooling module in frame aggregation, assessed the discriminative ability of the CA module, and compared the proposed BIF module with several common fusion strategies, thereby comprehensively assessing the performance and robustness of CABIF-Net under different modality conditions and task settings.

### 4.1. Experimental Environment and Parameter Settings

To ensure the reproducibility of experiments and consistency of performance comparison, all training and evaluation procedures were carried out in a unified software and hardware environment. The specific hardware configuration is detailed in Table 2. Additionally, the key hyperparameter settings for the experiments are summarized in Table 3.

### 4.2. Model Fine-Tuning Strategy

To make full use of the unique characteristics of the two datasets, a staged pre-training and fine-tuning strategy for the visual and audio encoders was designed to obtain reliable fine-grained bird representations. On the Birds21, the visual feature extraction was initialized using ImageNet pre-trained weights and fine-tuned on the training set to capture fine visual cues. The audio feature extraction was initialized with AudioSet pre-trained weights and also fine-tuned on the same training set to improve the ability to distinguish complex bird vocalizations.

On the SSW60, firstly, the visual feature extraction was fine-tuned on iNat2021, and then the obtained weights were used as the final visual parameters. The audio feature extraction also used AudioSet pre-trained weights for initialization and was fine-tuned using the unpaired audio set to obtain general representations of bird vocalizations, and then further fine-tuned on the original paired audio set to obtain the final weights. The above strategies are summarized in Table 4.

### 4.3. Overall Performance of CABIF-Net

To verify the performance of the proposed CABIF-Net in the fine-grained audio-visual bird classification task, several evaluation metrics such as accuracy, precision, recall, and F1 score were adopted on two datasets. The results are listed in Table 5. On the SSW60 with imbalanced modality and intensive noise, CABIF-Net achieved an accuracy of 85.76%, and demonstrated consistently superior performance in terms of precision, recall, and F1 score. On the Birds21 with more balanced modality, the method achieved an accuracy of 96.67%, indicating that the proposed fusion mechanism can not only effectively handle modality quality imbalance and noise interference in real-world field conditions, but also achieve higher discriminative performance under higher-quality data conditions.

### 4.4. Ablation Study

To evaluate the impact of key components in CABIF-Net on the final performance, six ablation experiments were conducted on the SSW60 and Birds21 test sets (the Top-K mean pooling module was not applied on Birds21 as it does not contain video frames). The experimental results are presented in Table 6.

Firstly, the performances of the individual visual and audio unimodal branches were evaluated. The SSW60 contains videos of 60 wild bird species, but due to the uncertainty of environment, the modality quality is imbalanced. Generally, visual data is relatively easy to acquire, while audio data is more susceptible to environmental noise and thus of lower quality. On SSW60, the accuracy of the visual unimodal was 81.32%, while that of the audio unimodal was only 33.00%. For Birds21, where the visual and audio modalities are relatively balanced and less affected by noise, the accuracies of visual-only and audio-only were 91.47% and 92.80%, respectively. This provides a baseline for analyzing the fusion performance under different modality quality conditions.

Subsequently, ablation experiments were carried out on the key modules of the model. The Concat fusion method, which directly concatenates multimodal features before classification, achieved an accuracy of 83.54% on SSW60 and 95.21% on Birds21. These results indicate that simple concatenation can improve performance, but it does not fully exploit cross-modal complementary information.

With the introduction of the BIF module, performance on Birds21 improved. This suggests that bidirectional inter-modulation can enhance information flow between different modalities. However, on SSW60, the performance of the BIF module was slightly inferior to Concat (about 0.21% lower), which might be due to the poor quality of the audio modality in SSW60. During the inter-modulation, the low-quality audio might interfere with the stronger visual modality, limiting the overall performance improvement.

When the CA module was integrated with BIF, the performance on both datasets was further improved. On Birds21, BIF+CA achieved the highest accuracy of 96.67%. On SSW60, the accuracy increased to 84.06%, which was approximately 0.48% higher than the Concat method. These results indicate that adjusting the modality contributions through sample-level confidence weights can suppress the interference of lower-quality modalities during the fusion process. On SSW60, further introducing the Top-K mean pooling (Top-K) module achieved an accuracy of 85.76%, demonstrating that selecting and aggregating discriminative key frames at the video level can effectively enhance discriminative features, significantly strengthen the video representation, and improve the overall classification performance. In summary, the ablation experiments show that each proposed component contributes to improving the fine-grained audio-visual fusion performance.

### 4.5. Comparison with Different Fusion Methods

To compare the performance of different fusion strategies in fine-grained audio-visual bird recognition, several fusion methods were evaluated on the SSW60 and Birds21 datasets. These fusion methods include Concat, logit-avg, shared-fusion, score-fusion, Cross-Attention, Multimodal Transformer, and the proposed CABIF-Net. To ensure a fair comparison on SSW60, the fusion methods that required visual aggregation at the clip-level adopted the Top-K mean pooling module to aggregate the visual features at the frame level, while Cross-Attention and Multimodal Transformer retained the multi-frame visual tokens according to their fusion characteristics. The quantitative results, reported as mean ± standard deviation over five runs, are shown in Table 7.

As shown in Table 7, on SSW60, the Concat method directly merges audio-visual features and achieved an accuracy of 84.66 ± 0.25%, while the shared-fusion method achieved an accuracy of 84.26 ± 0.07%. The Cross-Attention and Multimodal Transformer methods achieved accuracies of 82.81 ± 0.18% and 78.11 ± 0.38%, respectively. In contrast, accuracies of the logit-avg and score-fusion methods were 64.09 ± 0.00% and 72.01 ± 0.03%, respectively. This is because these methods mainly rely on posterior score combination or simplistic fusion strategies, lacking effective modeling of discriminative interactions within modalities and complementary information between modalities. In the case of modality quality imbalance in SSW60, high-quality features are prone to being interfered by low-quality modalities, resulting in a decline in overall classification performance.

On the benchmark Birds21, the quality of the visual and audio modalities is relatively balanced, and the performance differences among various fusion strategies are small. Among them, logit-avg and BIF+CA (CABIF-Net) performed better with accuracies of 95.32 ± 0.08% and 96.68 ± 0.06%, respectively. The Cross-Attention and Multimodal Transformer methods achieved accuracies of 94.55 ± 0.06% and 94.81 ± 0.23%, respectively. This further illustrates the influence of modality quality on the effectiveness of fusion strategy. Compared with the shared-fusion that only enhances the sharing of information flow between modalities, the proposed CABIF-Net further incorporates the CA module on top of the BIF module to quantify the reliability of each modality and dynamically adjust their contributions. This confidence-weighted mechanism can mitigate the negative impact of low-quality modalities on the fusion result, while preserving the discriminative information of high-quality modalities, enhancing the robustness and discriminative ability of the overall fusion. The mechanism is not a simple stack of modules, but a coordinated interaction among components, through which the collaborative improvement of fusion performance is achieved.

To further complement the comparison in Table 7, we also evaluated the variability, statistical significance and robustness under audio noise interference, and computational complexity of repeated runs. The detailed results can be found in Table A2, Table A3 and Table A4 in Appendix A. During repeated runs, CABIF-Net showed low variance on both datasets, indicating its stable performance across different random seeds. Moreover, after Holm adjustment, its improvements over the compared fusion baselines remained statistically significant. In the robustness experiments, Gaussian noise was added to the audio branch. On SSW60, CABIF-Net achieved the best original accuracy, and remained one of the best-performing methods even under noisy conditions. On Birds21, CABIF-Net also achieved the best original accuracy, while the simple Concat baseline was more robust under severe audio degradation. In terms of complexity, the overhead introduced by CABIF-Net on SSW60 was relatively small. However, on Birds21, it required higher FLOPs and latency than those of the simpler fusion baseline, although its number of parameters was still less than that of Cross-Attention and Multimodal Transformer.

**Figure 3:**
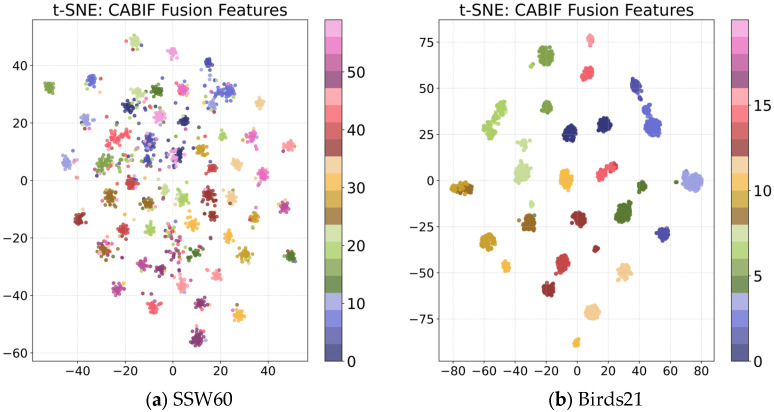
t-SNE visualization of fused feature embeddings on different datasets. (**a**) Feature distribution on the SSW60; (**b**) feature distribution on the Birds21. Each point represents a sample projected into a low-dimensional space, and different colors denote different bird species.

**Figure A1:**
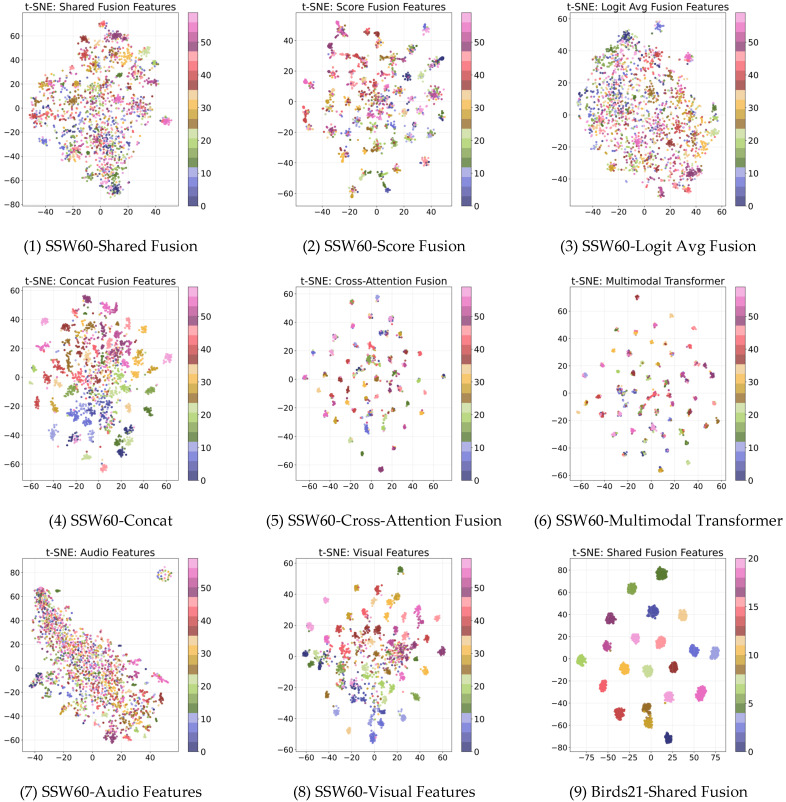

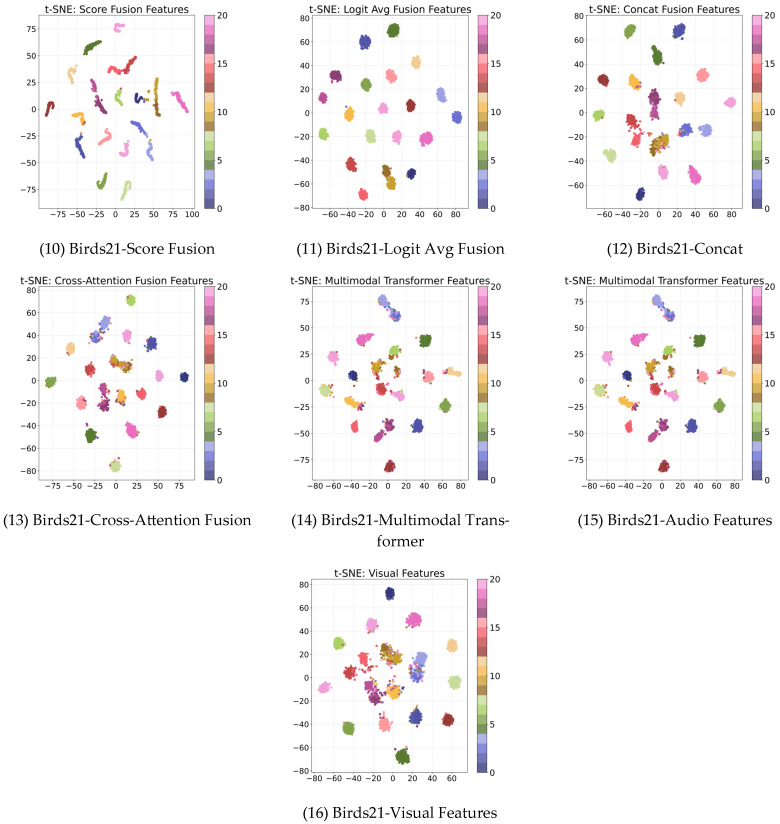
Visualization results of other fusion methods.

To further compare the effects of different fusion strategies at the feature representation level, the t-SNE dimensionality reduction visualization was performed on the features extracted through various fusion methods on the test sets, as shown in Figure 3. The visualization results of the other fusion methods are provided in Appendix A, Figure A1. These results clearly show that when using CABIF-Net, the features of the same class are clustered more closely and the distances between classes are larger, which reflects a stronger discriminative ability. As shown in Figure 3a, this phenomenon is particularly obvious on SSW60, indicating that through the integration of the Top-K mean pooling module, the CA module, and the BIF module, CABIF-Net more accurately evaluates the contributions of different quality modalities and performs targeted fusion, significantly improving classification performance and robustness.

These results validate the importance and necessity of the three key modules, namely the Top-K mean pooling module, the CA module, and the BIF module, in audio-visual modality fusion tasks.

### 4.6. Top-K Sensitivity Analysis

**Figure 4:**
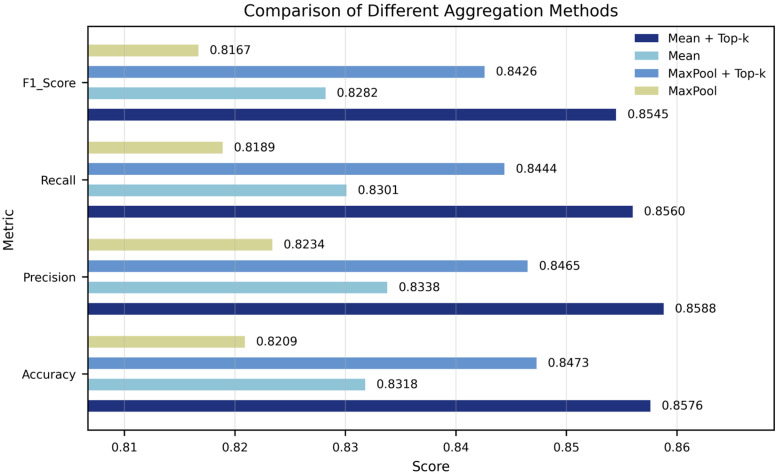
Comparison of Aggregation Methods on SSW60. The performance of mean pooling, max pooling, and their corresponding Top-K variants on SSW60 were compared. The results show that mean + Top-K achieved the highest scores in all metrics, indicating that Top-K effectively enhanced the discriminative ability of video frame aggregation.

To analyze the combined effect of Top-K with different frame aggregation strategies, this section first compares the performance of two frame aggregation methods, mean pooling and max pooling, combined with Top-K on the SSW60 dataset to determine the most suitable aggregation framework. As shown in Figure 4, without introducing the Top-K mechanism, the performance of mean pooling is superior to that of max pooling. With the Top-K mechanism, both mean pooling and max pooling have improved in terms of accuracy, precision, recall, and F1 score. Among them, mean+Top-K performs best in all indicators (accuracy: 0.8576, precision: 0.8588, recall: 0.8560, F1 score: 0.8545), significantly outperforming the aggregation scheme without introducing the Top-K. This indicates that the Top-K mechanism plays a crucial role in enhancing the discriminative ability of visual frames and reducing noise interference.

**Figure 5:**
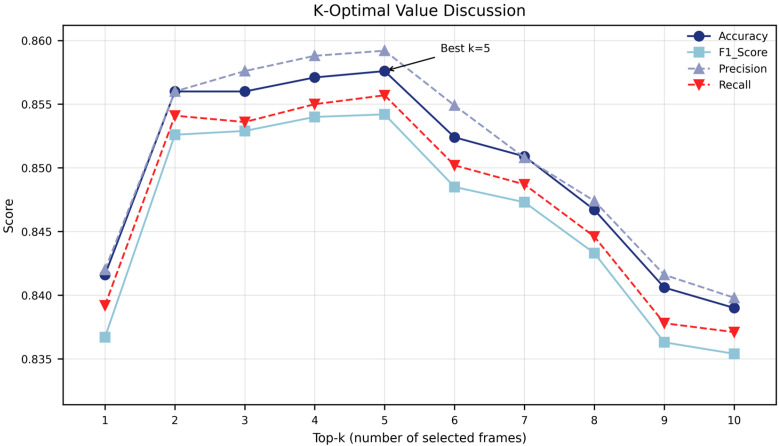
Top-K sensitivity analysis on SSW60 dataset. It shows the effect of different values of k (number of selected frames) on accuracy, precision, recall, and F1 score when using the mean pooling+Top-K aggregation strategy on the SSW60 test set. It is observed that the best performance is achieved when k is approximately equal to 5.

The impact of the parameter k on the performance was further examined. As shown in Figure 5, when k increases from 1 to 5, all indicators steadily rise and reach a peak at k = 5 (accuracy: 0.8576). When k exceeds 5, performance begins to decline. This suggests that too few key frames are insufficient to capture comprehensive discriminative information, while too many frames may introduce redundancy or noise, thereby weakening the discriminative effect. Considering both performance and computational cost, this study selects k = 5 as the empirical optimal setting for SSW60.

In summary, the introduction of the Top-K frame selection module significantly improves the discriminative ability of frame-level feature aggregation. Compared with simple mean or max pooling, the aggregation strategy combined with Top-K can more effectively select the most informative parts from redundant video frames, thereby enhancing the overall classification performance and robustness.

To validate the effectiveness of the proposed CA module in audio-visual fusion, this section analyzes the results from four perspectives: the overall weight distribution, class-level weight differences, the relationship between fusion confidence and weights, and representative sample analysis.

**Figure 6:**
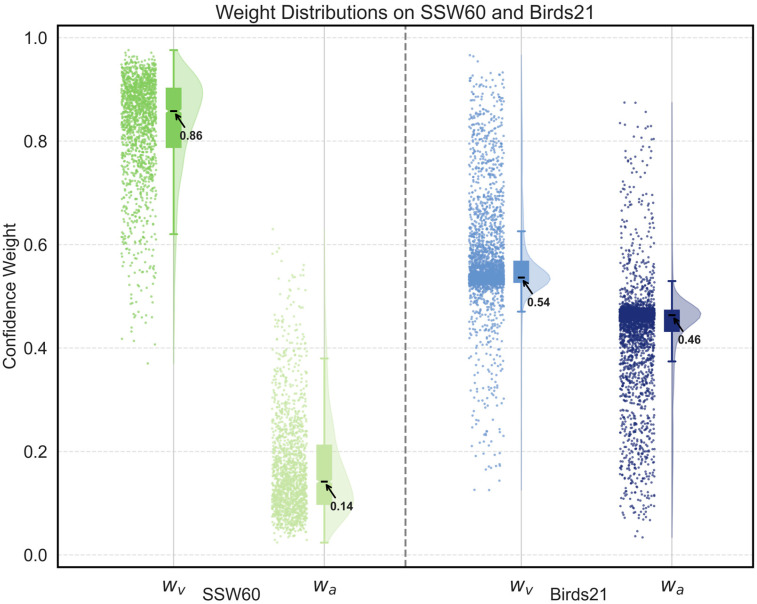
Weight distributions of SSW60 and Birds21. This raincloud plot combines density estimation, boxplots, and sample scatter to show distribution of visual modality weight $w_{v}$ and audio modality weight $w_{a}$ in all samples, revealing differences in the contributions of different modalities after CA.4.7 Modality Reliability Analysis.

The weight distribution reveals the reliability trend of each modality in all samples. Figure 6 shows raincloud plots of visual and audio weights of all samples in two datasets. On SSW60, the visual weights are higher than the audio weights, which is consistent with the fact that audio signals in natural environments are often disturbed by noise and contain less discriminative information. In contrast, on Birds21, the weight distributions of visual and audio are more balanced, reflecting that modality quality differences in this dataset are smaller, resulting in a more uniform weight assignment.

**Figure 7:**
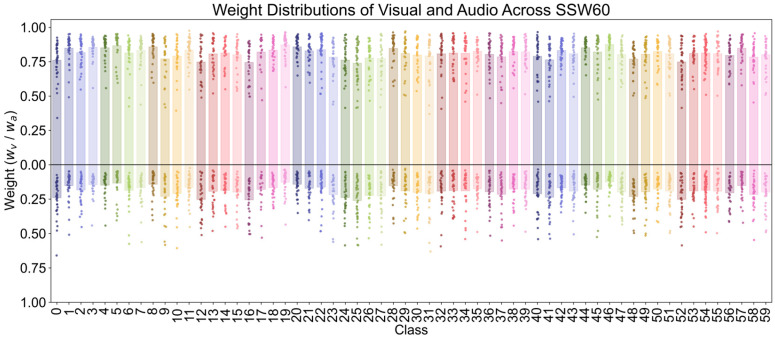
Class-wise weight distribution on SSW60. The combined average weight bar chart and weight scatter plot at a class level illustrate differences in visual and audio contributions of different categories in the SSW60.

**Figure 8:**
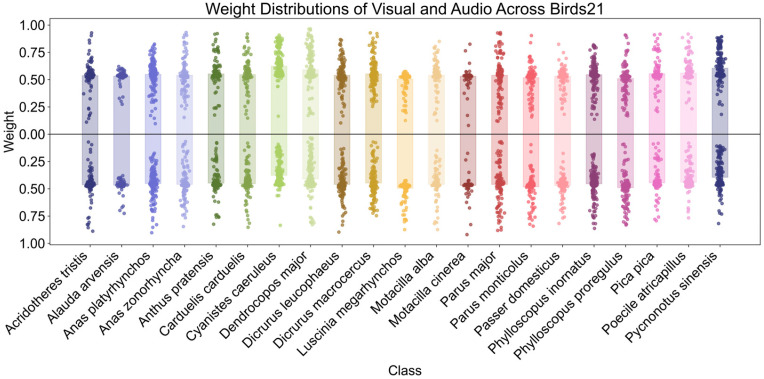
Class-wise weight distribution on Birds21. The average weight bar chart and weight scatter plot at the class level show the visual and audio contributions of different categories in the Birds21. Overall, the distribution is relatively balanced.

To further examine the trend at class-level, Figure 7 (SSW60) and Figure 8 (Birds21) show the distribution of visual and audio weights for each class, respectively. On SSW60, most categories show more concentrated and higher visual weights, while the audio weights are more dispersed. This indicates that the model is more inclined to rely more on the higher-quality visual modality during the fusion process. In contrast, on Birds21, the visual and audio weights of each category are relatively balanced, further demonstrating that the CA module can adaptively adjust weights according to the modality quality differences in different datasets.

**Figure 9:**
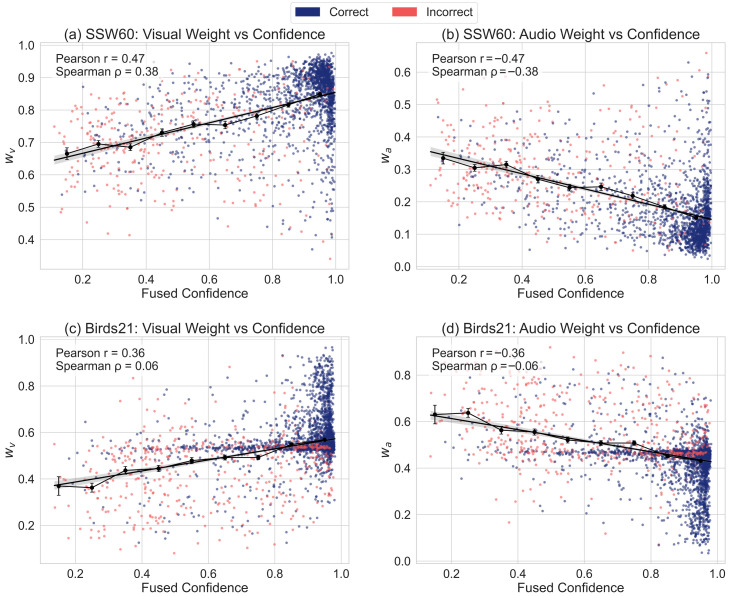
Relationship between confidence vs. modality weight. Each point represents a correctly predicted sample. The *x*-axis is the fused confidence, and the *y*-axis is the learned modal weight. The regression line and the trend of grouped (binned) average values visualize that higher fused confidence is usually in a direct proportion to higher modal weight contribution. The Pearson r and Spearman ρ are used to quantify linear correlation and rank correlations.

Figure 9 shows the relationship between the fused confidence and visual–audio weights. On SSW60, as the degree of confidence fusion increases, the visual weight shows a significant upward trend, while audio weight generally decreases. This indicates that when confidence is high, the model tends to amplify the contribution of the more reliable visual modality and diminish the influence of noisy audio modality. A similar but less obvious trend is also observed on Birds21, which is consistent with the relatively balanced quality of each modality in this dataset.

**Figure 10:**
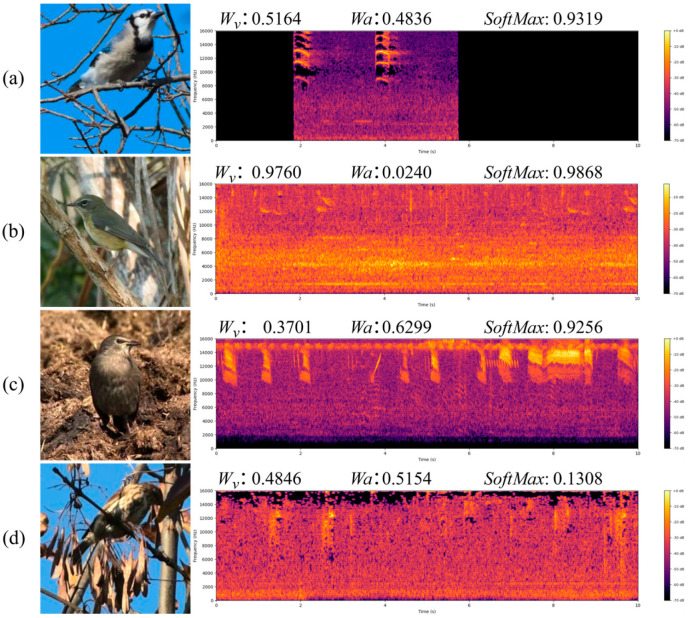
Visualization of representative cases (Image+Spectrogram). This figure shows four representative examples with image frames and corresponding audio spectrograms. In (**a**), the two modalities are clearly distinguishable, so the confidence of the fusion is high and the weight distribution is relatively balanced. In (**b**), the noisy audio causes the CA module to assign a higher weight to visual information. In (**c**), the weak visual cues cause the CA module to favor the audio. In (**d**), both modalities are very weak, resulting in a low confidence of fusion and misclassification. (All bird images in the figure are extracted from video frames of the SSW60 dataset).

To visually illustrate the effect of the CA module in discriminative fusion, Figure 10 shows four representative samples along with their corresponding visual images and audio spectrograms. In Figure 10a, both visual and audio information are relatively clear. The CA module assigns relatively balanced modality weights, indicating that both modalities are considered reliable and have similar contributions. In Figure 10b, the visual modality contains clear information sufficient to identify the bird species, while the audio modality has a large amount of background noise and no obvious bird vocalizations. In terms of weight assignment, the visual weight is significantly higher than the audio weight, which demonstrates that the CA module can reduce the influence of lower-quality modalities based on confidence. Figure 10c shows another case, where the audio modality contains clear bird vocalizations, while the visual modality performs poorly due to blending of the bird’s color with the background. In this case, the CA module assigns a higher weight to the audio modality and reduces the weight of the visual modality, so that the final fusion can better reflect the distinctive features of the audio signal. Figure 10d illustrates a sample in which neither visual nor audio modality provides effective discriminative features. Although the CA module adjusted the weights of these two modalities, due to insufficient input information, the fusion confidence remains low (0.1308) and the final classification result is incorrect. This indicates that the CA cannot generate new information, but in the case of limited information, it can prevent over-assigning weights to unreliable modalities, thereby reducing adverse effects. These representative cases show that the CA module can reasonably control the contribution of each modality based on confidence at the sample level, automatically reduce the weights of less reliable modalities, and enhance the robustness and reliability of discriminative fusion.

In summary, the CA module can dynamically evaluate the reliability of each modality based on the prediction confidence at the sample level, and accordingly adjust weight allocation, improving the overall classification performance and robustness. This mechanism is particularly evident in modality-imbalanced settings such as SSW60, but in datasets with relatively balanced modality quality, such as Birds21, the CA module can still finely adjust weights to enhance fusion performance.

## 5. Discussion

This study verifies the CABIF-Net on two datasets with different modality characteristics. The SSW60 represents a more challenging setting with stronger modality–quality imbalance and environmental noise. While Birds21 provides a comparatively balanced audio-visual setting at the species level, this contrast enables a clearer assessment of how CABIF-Net adapts to different modality conditions. Overall, the experimental results of the comparison between ablation and fusion indicate that the main advantage of CABIF-Net lies in modeling modality reliability at the sample level and achieving controllable cross-modal integration.

CABIF-Net was experimentally compared with the current State-of-the-Art bird recognition methods, and the results are shown in Table 8. On SSW60, Van Horn et al. utilized the score fusion strategy, which combined visual and audio outputs at the decision level, and achieved an accuracy of 80.60%, but the fusion process was mainly carried out at the decision level and did not explicitly model feature-level complementary information between different modalities [24]. Xu et al. introduced MMCosine loss with middle-layer connections on the ResNet-50 backbone network to alleviate the modality imbalance, yet this strategy still mainly relied on feature-level connections and did not provide an explicit modality weighting mechanism in noise perception or controllable cross-modal interaction [25]. The vision-based method proposed by Sun et al. achieved an accuracy of 70.1%, under conditions of environmental noise, occlusion, and background interference. This suggests that unimodal visual information may be insufficient to maintain a robust discrimination ability under challenging field conditions [37]. Xie et al. propose MSFG-AVFNet, which improved the quality of unimodal features through multi-stage fine-tuning. It also introduced an audio-visual loss to enhance modality consistency, and its accuracy on SSW60 reached 85.14% [26]. This suggests that improving representation quality and cross-modal consistency is an effective direction, but its fusion module still mainly relies on relatively fixed feature concatenation and aggregation, and lacks dynamic control of modality contributions when sample quality fluctuates significantly.

In contrast, the proposed CABIF-Net in this paper achieved an accuracy of 85.76% on SSW60, demonstrating its advantage in leveraging complementary audio-visual information for fine-grained classification. Specifically, CABIF-Net adopts a more robust fusion strategy in three aspects.

(1) The Top-K mean pooling module selects and aggregates the most reliable K frames based on confidence at the frame level, reducing the redundant low-quality frames on the video-level representations, significantly mitigating the negative effects of occlusion and background noise.
(2) The Confidence Calibration module explicitly maps the discriminative reliability of each modality to learnable and interpretable weights, effectively controlling the contribution of each modality in the case of quality imbalance, and preventing the noise of the weaker modality from degrading the stronger modality.
(3) The Bidirectional Inter-modulation Fusion module enables controllable complementary interaction through weights, allowing the stronger modality to dominate the interaction and incorporate the useful information of the weaker modality.

The above comparison results further indicate that the advantages of CABIF-Net not only lie in the improvement of performance, but also in its targeted modeling of environmental noise, modality quality fluctuation, and fine-grained inter-class confusion. On the SSW60 dataset, the samples were collected in real-world environments, which makes them more vulnerable to background noise, overlapping sounds, and changes in recording conditions. In this case, CABIF-Net does not simply combine the two modalities. Instead, it adopts a modality reliability-centered fusion mechanism. During the fusion process, it dynamically suppresses the influence of lower-quality modalities and strengthens the role of high-quality modalities, thereby improving the robustness of recognition under complex field conditions. Regarding the weights of the modalities, on the SSW60, the weights tend to favor the visual modality, while on Birds21 the weights are more balanced. This reflects the difference in modality quality between these two datasets. Moreover, the weight distribution at the class level shows that this trend is consistent in most categories. The relationship between fusion confidence and modality weights further suggests that the CA module can dynamically adjust the modality weights according to modality quality. Usually, when the audio modality is severely corrupted by noise or the visual modality is degraded by occlusion or camouflage, the fusion weights will shift toward the more reliable modality.

On the Birds21 dataset, the quality of the visual and audio modalities is relatively balanced. However, for some species, there are still similar appearance features or closely related vocalization patterns, which increases the difficulty of fine-grained discrimination. In such cases, CABIF-Net reduces inter-class confusion by integrating complementary discriminative cues from both modalities. When the acoustic evidence is ambiguous, visual information can provide additional discriminative cues. Conversely, when the visual evidence is weak, the audio modality can still provide complementary information. Through this cross-modal complementarity, the fused feature representations become more discriminative and help to alleviate the confusion caused by similar appearances or vocalizations. This trend is also reflected in the t-SNE visualizations. As shown in Figure 3 and Figure A1, the fused features of CABIF-Net exhibit more compact intra-class clustering and clearer inter-class separation. Overall, these findings indicate that CABIF-Net can not only suppress noise in complex environments, but also reduce confusion among similar species through the adaptive integration of complementary information.

These interpretations are further supported by repeated runs, robustness and complexity analyses. In multiple independent experiments, CABIF-Net has relatively low variance on both datasets. And after Holm adjustment, its improvements over the compared fusion baseline remained statistically significant, indicating that the observed benefits were stable rather than caused by random initialization. Under the condition of audio noise interference, CABIF-Net still performed the best on SSW60, which is consistent with its design for handling modality quality imbalance. On Birds21, CABIF-Net still achieved the best accuracy under original conditions. However, in severe audio degradation situations, simpler fusion strategies such as Concat were more robust. This suggests that stronger cross-modal interaction may not always be more advantageous, when one modality is heavily corrupted, because degraded information is more likely to propagate during the fusion process. In terms of computational cost, compared to Concat, the additional overhead of CABIF-Net is not significant. However, on Birds21, the computational cost of CABIF-Net becomes more noticeable, and its practical value should be weighed based on the specific deployment scenario. Overall, these findings indicate that when there is an imbalance in modality reliability but it has not completely collapsed, CABIF-Net performs particularly well. However, in cases of extreme degradation or stricter efficiency constraints, simpler fusion methods may be more applicable.

In multi-source field data such as camera trap videos and autonomous recording unit (ARU) audio, the absence or deterioration of a single modality is a common phenomenon [38]. Therefore, the proposed audio-visual adaptive fusion model in this study can provide support for the recognition of fine-grained bird species under complex field conditions.

Nevertheless, this study has some limitations. Firstly, modality reliability is indirectly characterized through model confidence, and in case of strong domain differences or systematic noise, its reliability may decrease. Secondly, using ResNet-18 as the backbone network may limit representational capacity. Thirdly, this study mainly focuses on species recognition performance and does not include downstream ecological analyses or ecological monitoring tasks. Fourthly, Birds21, as a supplementary reference dataset for SSW60, is mainly used to verify the effectiveness of the proposed method in a relatively controlled cross-modal environment. However, its visual and audio samples are only paired at the species level, rather than coming from the same observation event. Therefore, it cannot reflect real-world asynchronous or missing modalities. Future work can be conducted in the following aspects.

(1) Introduce more reliable uncertainty estimation and domain adaptation strategies to enhance cross-modal generalization.
(2) Explore stronger backbone networks and lightweight deployment schemes to improve accuracy while maintaining robustness and interpretability, so as to enable deployment on edge devices.
(3) Extend the proposed method to more realistic multimodal datasets and task settings to evaluate its applicability beyond bird species recognition.

## 6. Conclusions

This paper proposes a fusion framework, CABIF-Net, for fine-grained audio-visual bird classification, aiming to address issues such as modality quality imbalance and noise interference in real-world scenarios. The framework employs a confidence-based Top-K mean pooling module to select key frames and enhances representation quality at the video level. Then, the CA module is introduced to dynamically evaluate the reliability of visual and audio modalities. Finally, the BIF module is integrated at the feature level to achieve controllable information interaction. Experimental results show that CABIF-Net outperforms unimodal methods and various fusion strategies on SSW60, which has stronger noise interference and modality imbalance. Meanwhile, on the self-built Birds21, where the quality of the two modalities is relatively more balanced, CABIF-Net also performs well. These findings indicate that the proposed framework is effective for adaptive multimodal fusion under heterogeneous modality conditions. In addition, this framework can be easily integrated with other backbone networks and is applicable to other multimodal datasets and tasks. It provides an effective solution for robust multimodal recognition in ecological diversity monitoring.

## Figures and Tables

**Table 1:** Dataset description.

Dataset	Categories	Type	Samples	Train	Test	Source
SSW60	60	Video	5400	3462	1938	https://github.com/visipedia/ssw60 (accessed on 4 February 2026)
Birds21	21	Images	17,207	13,765	3442	https://www.macaulaylibrary.org/ (accessed on 4 February 2026)
		Audio	1186 recordings (total 76,477.8 s)	984	202	https://www.xeno-canto.org/ (accessed on 4 February 2026)

**Table 2:** The settings of experiments.

Test Environment	Type
System	Windows 10 64-bit
Graphics card	NVIDIA GeForce RTX A6000
CPU	AMD Ryzen 9 5920X @ 4.8 GHz
RAM	128 G
Framework	PyTorch 2.3.1
Programming language	Python 3.9 and MATLAB 2020a

**Table 3:** Hyperparameters of experiments.

Hyperparameter	Value
Optimizer	AdamW
Learning rate (fusion head & modulation MLPs)	3 × 10^−4^
Learning rate (confidence calibrator)	1 × 10^−2^
Weight decay	1 × 10^−4^
Batch size	64
Epochs	100
Label smoothing	0.02
Frame extraction	10 frames
Top-K	5

**Table 4:** Pre-training initialization, model architecture, and fine-tuning strategies for Birds21 and SSW60.

Dataset	Branch	Model	Initialization	Step 1 Fine-Tuning	Step 2 Fine-Tuning
Birds21	Visual	ResNet-18	ImageNet pretrained weights	Finetuned on Birds21 train	—
	Audio	ResNet-18	AudioSet pretrained weights	Finetuned on Birds21 train	—
SSW60	Visual	ResNet-18	ImageNet pretrained weights	Finetuned on iNat2021 images	Adopted as final weights
	Audio	ResNet-18	AudioSet pretrained weights	Finetuned on SSW60 unpaired audio (audio_ml)	Finetuned on SSW60 paired audio clips

**Table 5:** Overall classification performance of CABIF-Net on SSW60 and Birds21.

Dataset	Method	Acc (%)	Pre (%)	Recall (%)	F1 (%)
Birds21	CABIF-Net	96.67	96.49	96.37	96.39
SSW60		85.76	85.88	85.60	85.45

**Table 6:** Ablation study results showing the effects of individual components.

Dataset	Method	Acc (%)	Pre (%)	Recall (%)	F1 (%)
SSW60	Audio	33.00	32.90	33.10	31.60
	Image	81.32	81.73	81.08	80.96
	Concat	83.54	83.76	83.27	83.09
	BIF	83.23	83.48	83.07	82.78
	BIF+CA	84.06	84.05	83.92	83.59
	BIF+CA+Top-K	85.76	85.88	85.60	85.45
Birds21	Audio	92.80	91.00	91.60	91.30
	Image	91.47	91.09	90.89	90.94
	Concat	95.21	94.94	94.86	94.88
	BIF	95.65	95.42	95.28	95.30
	BIF+CA	96.67	96.49	96.37	96.39

**Table 7:** Comparison of different fusion strategies on SSW60 and Birds21 datasets.

Method	Dataset	Acc (%)	Pre (%)	Recall (%)	F1 (%)
Concat+Top-K	SSW60	84.66 ± 0.25	85.03 ± 0.26	84.56 ± 0.26	84.39 ± 0.29
shared-fusion+Top-K		84.26 ± 0.07	84.36 ± 0.09	84.05 ± 0.08	83.81 ± 0.04
logit-avg+Top-K		64.09 ± 0.00	66.49 ± 0.00	63.92 ± 0.00	63.70 ± 0.00
score-fusion+Top-K		72.01 ± 0.03	74.01 ± 0.03	71.78 ± 0.02	71.83 ± 0.03
Cross-Attention		82.81 ± 0.18	83.82 ± 0.44	82.57 ± 0.23	82.26 ± 0.10
Multimodal Transformer		78.11 ± 0.38	79.20 ± 0.35	77.88 ± 0.38	77.67 ± 0.32
BIF+CA+Top-K		85.58 ± 0.13	85.61 ± 0.18	85.39 ± 0.14	85.21 ± 0.17
Concat	Birds21	95.16 ± 0.10	94.88 ± 0.10	94.80 ± 0.12	94.82 ± 0.11
shared-fusion		95.19 ± 0.09	94.93 ± 0.08	94.79 ± 0.10	94.82 ± 0.09
logit-avg		95.32 ± 0.08	95.04 ± 0.09	94.97 ± 0.08	94.99 ± 0.09
score-fusion		92.91 ± 0.12	92.68 ± 0.07	92.48 ± 0.13	92.48 ± 0.13
Cross-Attention		94.55 ± 0.06	94.32 ± 0.06	94.15 ± 0.08	94.19 ± 0.07
Multimodal Transformer		94.81 ± 0.23	94.58 ± 0.25	94.42 ± 0.24	94.44 ± 0.24
BIF+CA		96.68 ± 0.06	96.50 ± 0.06	96.39 ± 0.06	96.41 ± 0.06

**Table 8:** Comparison with State-of-the-Art methods on SSW60.

Study	Fusion Method	Backbone	Loss Function	Accuracy (%)
Van Horn et al. [24]	Score fusion	VIT-B	Cross-entropy loss	80.6
Xu et al. [25]	Mid concatenation	ResNet-50	MMCosine loss	75.95
Sun et al. [37]	-	Visual: VideoPrism	Cross-entropy loss	70.1
Xie et al. [26]	Mid concatenation	Visual: YOLOv11, Audio: CNN14	AVloss	85.14
Proposed work	Feature fusion	ResNet-18	Cross-entropy loss	85.76

## Data Availability

All data included in this study are available from the corresponding author upon reasonable request. Public datasets used in this work are available from the following sources: Xeno-canto bird sound archive (https://xeno-canto.org/, accessed on 4 February 2026), the Macaulay Library media repository (https://www.macaulaylibrary.org/, accessed on 4 February 2026), and the SSW60 dataset at https://github.com/visipedia/ssw60 (accessed on 4 February 2026). The source code for CABIF-Net is publicly available at https://github.com/dragonlong107/CABIF-Net (accessed on 19 March 2026).

## Appendix A

**Table A1:** Species composition, source distribution, and sample statistics of the Birds21 dataset.

ID	Scientific Name	Genus	Family	Taxon Code	Image Geographic Scope	Audio Geographic Scope	No. of Images	No. of Audio
1	*Anas platyrhynchos*	*Anas*	*Anatidae*	mallar3	Europe	Europe	576	55
2	*Anas zonorhyncha*	*Anas*	*Anatidae*	spbduc	East Asia	East Asia	541	53
3	*Dicrurus leucophaeus*	*Dicrurus*	*Dicruridae*	ashdro1	East Asia	East Asia	403	57
4	*Dicrurus macrocercus*	*Dicrurus*	*Dicruridae*	bladro1	East Asia	East Asia	550	55
5	*Parus major*	*Parus*	*Paridae*	gretit1	Europe	Europe	460	52
6	*Parus monticolus*	*Parus*	*Paridae*	grbtit1	East Asia	East Asia	591	53
7	*Phylloscopus inornatus*	*Phylloscopus*	*Phylloscopidae*	yebwar3	Europe	East Asia&Europe	550	67
8	*Phylloscopus proregulus*	*Phylloscopus*	*Phylloscopidae*	palwar5	East Asia	East Asia	531	74
9	*Passer domesticus*	*Passer*	*Passeridae*	houspa	Europe	Europe	1023	53
10	*Pica pica*	*Pica*	*Corvidae*	eurmag1	Europe	Europe	865	55
11	*Acridotheres tristis*	*Acridotheres*	*Sturnidae*	commyn	East and South Asia	East and South Asia	990	55
12	*Motacilla cinerea*	*Motacilla*	*Motacillidae*	grywag	Europe	Europe	1046	60
13	*Motacilla alba*	*Motacilla*	*Motacillidae*	whiwag	Europe	Europe	1049	59
14	*Alauda arvensis*	*Alauda*	*Alaudidae*	skylar	Europe	Europe	818	55
15	*Anthus pratensis*	*Anthus*	*Motacillidae*	meapip1	Europe	Europe	999	55
16	*Luscinia megarhynchos*	*Luscinia*	*Muscicapidae*	comnig1	Europe	Europe	1047	55
17	*Poecile atricapillus*	*Poecile*	*Paridae*	bkcchi	North America	North America	1053	55
18	*Cyanistes caeruleus*	*Cyanistes*	*Paridae*	blutit	Europe	Europe	1024	54
19	*Dendrocopos major*	*Dendrocopos*	*Picidae*	grswoo	Europe	Europe	1033	54
20	*Carduelis carduelis*	*Carduelis*	*Fringillidae*	eurgol	Europe	Europe	1002	55
21	*Pycnonotus sinensis*	*Pycnonotus*	*Pycnonotidae*	livbul1	East Asia	East Asia	1056	55

**Table A2:** Repeated-run performance variability and statistical significance analysis of CABIF-Net on SSW60 and Birds21.

Comparison	Dataset	Metric	CABIF-Net (%)	Baseline (%)	Mean Diff. (%)	95% CI of Diff. (%)	p (Raw)	p (Holm-Adjusted)	$\mathrm{Cohen}’s\;d_{z}$
CABIF-Net vs. Concat+Top-K	SSW60	Acc	85.58 ± 0.13	84.66 ± 0.25	0.92	[0.65, 1.19]	0.000735	0.001469	4.17
		F1	85.21 ± 0.17	84.39 ± 0.29	0.82	[0.43, 1.22]	0.004322	0.004322	2.61
CABIF-Net vs. shared-fusion+Top-K		Acc	85.58 ± 0.13	84.26 ± 0.07	1.32	[1.19, 1.45]	0.00001	0.000072	12.35
		F1	85.21 ± 0.17	83.81 ± 0.04	1.4	[1.22, 1.58]	0.00003	0.000148	9.45
CABIF-Net vs. logit-avg+Top-K		Acc	85.58 ± 0.13	64.09 ± 0.00	21.5	[21.34, 21.66]	<0.000001	<0.000001	165.98
		F1	85.21 ± 0.17	63.70 ± 0.00	21.51	[21.30, 21.72]	<0.000001	<0.000001	125.94
CABIF-Net vs. score-fusion+Top-K		Acc	85.58 ± 0.13	72.01 ± 0.03	13.57	[13.38, 13.76]	<0.000001	<0.000001	90.21
		F1	85.21 ± 0.17	71.83 ± 0.03	13.38	[13.15, 13.61]	<0.000001	<0.000001	71.89
CABIF-Net vs. Cross-Attention		Acc	85.58 ± 0.13	82.81 ± 0.18	2.78	[2.46, 3.09]	0.000017	0.000101	10.9
		F1	85.21 ± 0.17	82.26 ± 0.10	2.95	[2.71, 3.19]	0.000005	0.000036	15.16
CABIF-Net vs. Multimodal Transformer		Acc	85.58 ± 0.13	78.11 ± 0.38	7.47	[6.99, 7.96]	0.000002	0.000024	19.17
		F1	85.21 ± 0.17	77.67 ± 0.32	7.54	[7.08, 8.01]	0.000001	0.000022	20.2
CABIF-Net vs. Concat	Birds21	Acc	96.68 ± 0.06	95.16 ± 0.10	1.52	[1.40, 1.63]	0.000003	0.000033	16.29
		F1	96.41 ± 0.06	94.82 ± 0.11	1.59	[1.47, 1.71]	0.000003	0.000033	16.39
CABIF-Net vs. shared-fusion		Acc	96.68 ± 0.06	95.19 ± 0.09	1.49	[1.40, 1.58]	0.000001	0.00002	20.97
		F1	96.41 ± 0.06	94.82 ± 0.09	1.59	[1.49, 1.70]	0.000002	0.000024	19.24
CABIF-Net vs. logit-avg		Acc	96.68 ± 0.06	95.32 ± 0.08	1.36	[1.27, 1.46]	0.000002	0.000027	18.04
		F1	96.41 ± 0.06	94.99 ± 0.09	1.42	[1.32, 1.52]	0.000002	0.000027	17.76
CABIF-Net vs. score-fusion		Acc	96.68 ± 0.06	92.91 ± 0.12	3.77	[3.61, 3.93]	<0.000001	0.000006	29.04
		F1	96.41 ± 0.06	92.48 ± 0.13	3.93	[3.74, 4.11]	<0.000001	0.000009	26.28
CABIF-Net vs. Cross-Attention		Acc	96.68 ± 0.06	94.55 ± 0.06	2.13	[2.06, 2.19]	<0.000001	0.000002	41.5
		F1	96.41 ± 0.06	94.19 ± 0.07	2.22	[2.13, 2.31]	<0.000001	0.000005	30.49
CABIF-Net vs. Multimodal Transformer		Acc	96.68 ± 0.06	94.81 ± 0.23	1.88	[1.56, 2.19]	0.000083	0.000311	7.29
		F1	96.41 ± 0.06	94.44 ± 0.24	1.97	[1.64, 2.30]	0.000078	0.000311	7.41

**Table A3:** Robustness comparison of different fusion methods under audio noise corruption on SSW60 and Birds21, reported in accuracy (%).

Method	Dataset	Origin	20 dB	15 dB	10 dB	0 dB	Drop@0 dB
Concat+Top-K	SSW60	84.98	83.49	81.94	80.91	80.44	4.54
shared-fusion+Top-K		84.26	83.38	82.82	81.94	49.59	34.67
logit-avg+Top-K		64.09	64.14	59.39	55.93	60.01	4.08
score-fusion+Top-K		72.03	73.22	71.05	69.61	77.09	−5.06
Cross-Attention		82.51	81.73	81.27	80.34	58.1	24.41
Multimodal Transformer		79.26	77.4	76.68	75.13	73.01	6.24
CABIF-Net		85.76	84.11	82.71	81.22	81.17	4.59
Concat	Birds21	95.53	90.77	88.4	85.84	85.41	10.12
shared-fusion		95.66	91.21	71.02	25.63	12.85	82.8
logit-avg		95.22	92.18	80.48	27.47	5.16	90.06
score-fusion		93.02	90.88	43.8	8.51	4.34	88.68
Cross-Attention		94.53	91.31	69.84	14.49	4.34	90.19
Multimodal Transformer		94.91	89.98	76.64	59.65	47.53	47.38
CABIF-Net		96.68	84.64	71.61	56.45	53.23	43.44

**Table A4:** Computational complexity comparison of different fusion methods on SSW60 and Birds21.

Method	Dataset	Params (M)	FLOPs (G)	Latency (ms)
Concat+Top-K	SSW60	54.4747	98.8507	18.346
shared-fusion+Top-K		54.7345	98.8512	17.6977
logit-avg+Top-K		54.4101	98.8506	16.1527
score-fusion+Top-K		27.2051	49.4255	10.4198
Cross-Attention		56.5744	98.8517	17.0902
Multimodal Transformer		58.6603	98.8759	17.4607
CABIF-Net		55.0005	98.8512	17.6686
Concat	Birds21	27.1996	20.9796	9.6465
shared-fusion		27.7224	20.9801	8.9136
logit-avg		27.1966	20.9795	8.1179
score-fusion		27.1966	20.9795	9.3755
Cross-Attention		31.4021	20.9816	10.5937
Multimodal Transformer		31.396	20.9859	10.3345
CABIF-Net		27.7255	41.9597	18.1501
